# The Relationship between Physical Fitness and Simulated Firefighting Task Performance

**DOI:** 10.1155/2018/3234176

**Published:** 2018-04-12

**Authors:** Goris Nazari, Joy C. MacDermid, Kathryn E. Sinden, Tom J. Overend

**Affiliations:** ^1^Health & Rehabilitation Science, Physiotherapy, Western University, London, ON, Canada; ^2^Roth McFarlane Hand and Upper Limb Centre, St. Joseph's Hospital, London, ON, Canada; ^3^School of Kinesiology, Lakehead University, 955 Oliver Road, Thunder Bay, ON, Canada

## Abstract

The overall aim of this study was to measure the physiological responses of firefighters from a single fire service during simulated functional firefighting tasks and to establish the relationship between physical fitness parameters and task performance. 46 males and 3 females firefighters were recruited. Firefighters' aerobic capacity levels were estimated using the Modified Canadian Aerobic Fitness Test (mCAFT). Grip strength levels, as a measure of upper body strength levels, were assessed using a calibrated J-Tech dynamometer. The National Institute for Occupational Safety and Health (NIOSH) protocol for the static floor lifting test was used to quantify lower body strength levels. Firefighters then performed two simulated tasks: a hose drag task and a stair climb with a high-rise pack tasks. Pearson's correlation coefficients (*r*) were calculated between firefighters' physical fitness parameters and task completion times. Two separate multivariable enter regression analyses were carried out to determine the predictive abilities of age, sex, muscle strength, and resting heart rate on task completion times. Our results displayed that near maximal heart rates of ≥88% of heart rate maximum were recorded during the two tasks. Correlation (*r*) ranged from −0.30 to 0.20. For the hose drag task, cardiorespiratory fitness and right grip strength (kg) demonstrated the highest correlations of −0.30 and −0.25, respectively. In predicting hose drag completion times, age and right grip strength scores were shown to be the statistically significant (*p* < 0.05) independent variables in our regression model. In predicting stair climb completion times, age and NIOSH scores were shown to be the statistically significant (*p* < 0.05) independent variables in our regression model. In conclusion, the hose drag and stair climb tasks were identified as physiological demanding tasks. Age, sex, resting heart rate, and upper body/lower body strength levels had similar predictive values on hose drag and stair climb completion times.

## 1. Introduction

Firefighters are not only set to fight fire but also respond to emergency situations to search, rescue, and protect health and community property [1, 2]. It is well documented that firefighting is a dangerous and physiologically demanding profession [3]. The most recent statistics in the United States reported that 51% of firefighter deaths in 2015 were due to sudden cardiac arrests [4]. Potential contributing factors are carrying heavy equipment with protective gear, heat stress, and working at near maximal heart rates for extended periods of times [2]. It is rather difficult and complex to quantify the actual physiological demands during real firefighting situations [5]. However, several studies have assessed firefighters' physiological responses (heart rate, percentage of maximum heart rate, rating of perceived exertions, and oxygen uptake) during simulated firefighting tasks (ceiling overhaul, stair climb with a high-rise pack, crawl, search, and rescue) [6].

Numerous studies have demonstrated a direct association between better firefighting job performance with higher levels of physical fitness [7]. Two aspects of fitness, cardiorespiratory and muscle strength, have received a great deal of attention. Cardiorespiratory fitness is an essential contributing factor to improved performance and enables firefighters to carry out on-duty tasks more efficiently [8]. Most recently, Kales et al. (2007) reported a >45 mL/kg/min levels of cardiorespiratory fitness in order for firefighters to safely and successfully complete on-duty tasks [9]. Furthermore, higher upper body muscle strength levels have been shown to demonstrate substantial correlation with faster task completion times [7].

There is growing body of literature that aims to quantify the physiological demands and establish the relationships between physical fitness parameters and simulated firefighting task performance. However, there is a paucity of reports in the literature regarding the physiological demands and the associations between physical fitness with simulated task performance, among Canadian firefighters. This study aimed to explore the extent to which Canadian firefighters' physiological responses and physical fitness measures would correspond with the US and European firefighters during simulated firefighting tasks. It was hypothesized that parameters such as cardiorespiratory fitness and upper and lower body strength levels would demonstrate negative correlations with the individual simulated functional task completion times [7].

Therefore, the overall objective of this study was to measure the physiological responses of Canadian firefighters from a single fire service during simulated functional firefighting tasks and to establish the relationship between physical fitness parameters and task performance. The specific goals were to determine (1) firefighters' physiological responses during two simulated functional tasks, hose drag and stair climb with a high-rise pack; (2) relationship between firefighters' physical fitness parameters (cardiorespiratory fitness and muscle strength) and simulated functional task completion times, (3) whether age, sex, fitness parameters, and/or physiological measures could be used to predict functional task completion times in firefighters.

## 2. Material and Methods

### 2.1. Study Design

We first explained the study purpose and procedures to each firefighter, who was then required to complete the PAR-Q and sign a Consent Form. The screening procedures were then carried out including the measures of resting heart rate (beats/minute), height in meters (m), and body weight (kg). Next was the fitting of Zephyr BioHarness and administering of the mCAFT. Once the mCAFT was terminated, firefighters were asked to be seated for a period of four and half minutes as recommended by the mCAFT protocol [10], and then we proceeded with the muscle strength level assessments. Standardized procedures were carried out when administering the mCAFT and assessing the upper and lower body strength levels. Firefighters were then asked to put on their full personal protective equipment and self-contained breathing apparatus. The simulated firefighting tasks, first the hose drag and then the stair climb with a high-rise pack, were performed at the end.

### 2.2. Measure of Fitness Parameters

#### 2.2.1. Cardiorespiratory Fitness

We followed the Modified Canadian Aerobic Fitness Test's (mCAFT) protocol to obtain an estimated maximal oxygen consumption (VO_2-max_ mL/kg/min) levels [10]. Each firefighter was required to complete a number of progressively demanding three-minute stepping sessions at a rate determined by a metronome assigned based on stage corresponding to their age and sex [10]. Firefighters were required to carry out as many of these three-minute stepping sessions until 85% of their maximum heart rate was achieved [85% maximum heart rate was estimated based on age-predicted equation (HR_max_ = 220 − age)]. The mCAFT was considered complete, once the 85% heart rate mark was achieved [11]. Firefighters' estimated VO_2-max_ (mL/kg/min) levels were calculated using the mCAFT's formula: 17.2 + (1.29 × oxygen cost at the final stage) − (0.09 × weight in kg) − (0.18 × age in years). The mcAFT is deemed as a reliable and valid submaximal test in estimating VO_2-max_ (mL/kg/min) levels [12].

#### 2.2.2. Upper Body Strength

Grip strength was measured using a calibrated J-Tech dynamometer. Firefighters were instructed to squeeze the device as hard as possible using the standardized positioning with elbow flexed at 90 degrees and forearm in neutral position while seated [13]. The same procedure was again repeated on the opposite hand. Grip strength levels for the right, left, and combined (right and left) hands were reported in kilograms (kg).

#### 2.2.3. Lower Body Strength

The National Institute for Occupational Safety and Health (NIOSH) protocol for the static floor lifting test was used to quantify lower body strength [11]. Firefighters were instructed to stand with feet shoulder width apart and the middle of the ankles at the mark on the platform while holding the handle with the back straight and knees and legs bent. The firefighters were then asked to pull upward with their best effort for 10 seconds. Two trials were carried out with a 30-second rest in between [11]. The lifting scores were reported were in kilograms (kg).

#### 2.2.4. Quantifying Physiological Responses

We used a reliable and valid device, Zephyr BioHarness™ (Zephyr Technology Corporation, Annapolis, MD, US), to quantify physiological responses [14]. Zephyr BioHarness and its wireless Bluetooth application connected to our laptop were used to monitor, capture, and transmit firefighters' physiological responses including heart rate (in absolute, beats/minute; relative, percentage of maximum heart rate, terms) and respiratory rates (breaths/minute), both at 2.5-second interval during the simulated firefighting tasks and the mCAFT. The rating of perceived exertion (RPE) was also assessed after completing each of the simulated firefighting tasks using a modified 10-point Borg scale. Firefighters were asked to rate on a scale of 0–10, where 0 was “like nothing at all” and 10 was “the most difficulty,” how difficult the simulated tasks were to perform [15].

### 2.3. Sampling and Recruitment

Ethical approval was secured for this study through the Hamilton Integrated Research Ethics Board. We randomly selected a total of *n* = 49 (46 males, 3 females) firefighters, (*n* = 49: recruits = 5; volunteer = 2; professional = 22; 1st rank firefighter = 10; captain = 10) from the complete sample of active duty firefighters (*n* = 330), Ontario, Canada. Male and female firefighters between 20–69 years of age, with the ability to read, write, and communicate in English, and those with “no” answers to all seven self-reported Physical Activity Readiness Questionnaire (PAR-Q) questions were included in this study [10]. Firefighters with response/s “yes” to any of seven PAR-Q questions were excluded from the study [10].

### 2.4. Simulated Firefighting Tasks

The hose drag and stair climb with a high-rise pack tasks are included in firefighters' standard entry level test. Firefighters travelled to the City of Hamilton firefighting training facility. This granted access to bona fide firefighting equipment and allowed firefighters to perform both the simulated tasks in full personal protective equipment (22.7 kg) and the self-contained breathing apparatus (SCBA) (18.1 kg).

#### 2.4.1. Hose Drag

A designated start/finish line was established from which each task was initiated and terminated. The hose drag task required firefighters to begin in standing, at the designated start/finish position. When instructed to begin, the firefighters bent to floor level and lifted the nozzle (6.10 kg.) of an uncharged firefighting hose (30 m). The firefighters were given standardized instructions to pull the uncharged fire hose a distance of 18 m to a pylon. Once at the pylon, the firefighters maneuvered the hose around the pylon at 90 degrees and pulled the hose to an end marker (12 m from the pylon). The firefighters then repeated the task while returning the nozzle to the start/finish line [16, 17]. Firefighters were timed while performing the task using a stopwatch.

#### 2.4.2. Stair Climb with a High-Rise Pack

Firefighters retrieved and lifted a high-rise pack (18.1 kg) from floor level to their preferred shoulder. The high-rise pack was contained with a green strap and included two links of hose and a nozzle. Once the firefighters stabilized the high-rise pack on their preferred shoulder, they ascend and descend stairs to the 4th floor of the training facility (112 total steps) [16, 17]. Firefighters were again timed while performing the task using a stopwatch.

Firefighter were asked to carry out both the simulated tasks at a work rate typically utilized at a fire scene.

### 2.5. Statistical Analysis

Analyses were performed using IBM SPSS Statistics software version 22.0 and a significance level of *p* ≤ 0.05 was considered statistically significant. Anthropometrics, fitness, and physiological responses of firefighters during the two simulated tasks were presented using means, standard deviations, and maximum and minimum scores. Pearson's correlation coefficients (*r*) were calculated between firefighters' physical fitness parameters and simulated functional task completion times. The strength of correlation (*r*) as proposed by Evans 1996 was <0.10 “trivial,” 0.20–0.39 “weak,” 0.40–0.59 “moderate,” 0.60–0.79 “strong,” and 0.80–1.00 “very strong” [18]. To predict functional task completion times (our dependent variable) in firefighters, we conducted two separate multivariable enter regression analyses: one for hose drag task and the second for the stair climb with a high-rise pack task. For the hose drag task, our model included age, sex, grip strength, and resting heart rate as the independent variables. For the stair climb with a high-rise pack task, our model included age, sex, NIOSH, and resting heart rate as the independent variables. All the assumptions of multiple regression being the tests of normality, heteroscedasticity, multicollinearity, and linearity were met prior to our regression analysis.

## 3. Results

### 3.1. Sample/Physiological Responses

Table 1 presents the male, female, and all firefighter demographic characteristics while the physiological responses during simulated functional tasks are displayed in Table 2. The Pearson's correlation coefficients for firefighters' fitness parameters (cardiorespiratory fitness and muscle strength levels) and each simulated functional task are presented in Table 3. The coefficients ranged from −0.30 to 0.20. The results indicated that the performance on simulated functional tasks, based on the time of completion, was related to fitness parameters. Negative Pearson's correlation coefficients indicated that higher VO_2max_ and/or strength levels were associated with faster completion of tasks and, therefore, better firefighting performance. For the hose drag task, cardiorespiratory fitness (VO_2max_ mL/kg/min) and right grip strength (kg) demonstrated the highest correlations of −0.30 and −0.25 (weak), respectively. For the stair climb with a high-rise pack task, only cardiorespiratory fitness (VO_2max_ mL/kg/min), with a correlation coefficient of −0.31, was associated with faster completion times and thus higher performance. In predicting hose drag completion times, firefighters' age and right grip strength scores were shown to be the two statistically significant (*p* < 0.05) independent variables in our multivariable regression model (Table 4). Sex, resting heart rate, and left grip strength measures were not statistically significant. Together, age, grip strength, sex, and resting heart rate accounted for 24% of the variance in firefighters' hose drag completion times. With 1-year increase in age, hose drag completion time increases by 0.48 seconds. With 1-point increase in right grip strength score, hose drag completion time decreases by 0.77 seconds. In predicting stair climb with a high-rise pack completion times, firefighters' age and NIOSH scores were shown to be the two statistically significant (*p* < 0.05) independent variables in our multivariable regression model (Table 5). Sex and resting heart rate measures were not statistically significant. Together, age, NIOSH, sex, and resting heart rate accounted for 25% of the variance in firefighters' stair climb completion times. With 1-year increase in age, stair climb completion time increases by 0.46 seconds. With 1-point increase in NIOSH score, stair climb completion time increases by 0.21 seconds.

## 4. Discussion 

This study found that the simulated hose drag and stair climb with a high-rise pack tasks were physiologically demanding, requiring firefighters to work at near maximal heart rates. Higher cardiorespiratory fitness levels were associated with better firefighting task performance, whereas upper and lower body strength levels were only associated with higher hose drag task performance. In addition, age, sex, resting heart rate, and upper body/lower body strength levels had similar and moderate predictive values in firefighting task completion times.

It is difficult and too complex to quantify firefighters' actual physiological demands during real firefighting situations [19]. Therefore, we monitored their physiological responses during two simulated functional tasks, hose drag and stair climb with a high-rise pack. These simulated tasks are included in Candidate Physical Ability Test, firefighters' standard entry level test, and are similar to regular on-duty tasks performed [16, 17]. Our findings suggested that firefighters worked at near maximal heart rates of 88% (hose drag) and 89% (stair climb), despite low ratings of perceived extortions. We were unable to compare our physiological response results with the firefighting relevant studies due to (1) wide range of protocol differences noted and (2) reporting of cumulative physiological scores with four to five simulated tasks performed in sequences.

Understanding physical fitness contribution with respect to firefighting task completion times would provide firefighters with new insights into more efficient and proper training strategies. Therefore, we aimed to establish the relationships between cardiorespiratory fitness and muscle strength levels on both the simulated functional tasks. We found that higher cardiorespiratory fitness levels were associated with faster performances on both the hose drag and stair climb tasks with correlation coefficients of −0.30 and −0.31, respectively. Our coefficients corresponded well with Myhre et al. (1997) study of 218 male and 4 female firefighters (mean age of 30.4 years; VO_2max_ = 39.4 mL/kg/min), which reported that higher performance on a rescue task was associated with higher cardiorespiratory fitness levels *r* = −0.36 [20]. In regard to the hose drag task, our results displayed that higher upper and lower body strength levels were associated with faster performance. Our findings corresponded well with previous studies. Myhre et al. (1997) reported that the correlations between upper body strength levels (bench press) with rescue task completion time was *r* = −0.18 [20]. Michaelides et al. (2011) study of 67 male firefighters (mean age of 33 years) displayed correlations of *r* = −0.22 between grip strength and a hose advance task [7]. The same study reported correlations of *r* = −0.26 between lower body strength levels (squat 1-repetition maximum) and a hose drag task [7]. On the contrary, Rhea et al. (2004) study of 17 male and 3 female fighters (mean age of 34.5 years) displayed correlation of −0.85 between grip strength levels and the hose drag task [21]. The higher correlations reported may be attributed to procedural difference in how the grip strength was measured (sitting versus standing). In regard to the stair climb task, our results displayed that higher upper and lower body strength levels were associated with slower performance. Similarly, our findings corresponded well with Michaelides et al. (2011), which displayed correlations of *r* = 0.16 between grip strength and a stair climb task [7]. In addition, lower body strength levels (squat 1-repetition maximum), displayed correlations of −0.02 with a stair climb task [7]. Overall, our results demonstrated correlations ranging from −0.20 to 0.20 between muscle strength levels and simulated firefighting task performance. A possible explanation for such weak correlations might be better understood by exploring the relationship between muscle strength and agility. Agility is defined as one's ability to change direction or start and stop, rapidly performing a complex motor activity [22]. Studies carried out among healthy participants/athletes have shown low correlations between muscle strength levels and agility tests [22, 23]. Although the simulated firefighting tasks utilized in our study were not agility tests, the tasks did include certain elements that would normally comprise an agility test, rapid start and stopping and change of directions and coordination.

As the Canadian population ages, maintaining work ability within physically demanding professions such as firefighting presents new challenges [24]. Studies have also reported that muscle strength levels among firefighters have been shown to improve task efficiency and performance and remain unchanged with aging [8, 25]. Furthermore, male and female firefighters are required to perform similarly on-duty tasks, despite qualitative study reports suggesting strength limitations of female firefighters when compared to their male counterparts [26]. Therefore, we aimed to create a regression model that would could be used to predict firefighting task completion times. Our first multiple regression analyses demonstrated that age and right grip strength levels were significant variables, and, with addition of left grip, sex, and resting heart rate, our model displayed a 24% predictive value for the hose drag performance task. Our second multiple regression analyses demonstrated that age and NIOSH scores (lower body strength) were both significant variables, and, with addition of sex and resting heart rate, our model displayed a 25% predictive value for the stair climb performance task. Sex was not identified as a significant variable in predicting task performance. This could be due to inclusion of only 3 female fighters, hence a power issue.

We assessed cardiorespiratory fitness levels by administering the mCAFT which is a submaximal test. We did this because it is a standardized test with a potential for widespread application. It is possible that submaximal testing is less accurate than maximal testing protocols; however, submaximal testing requires minimal equipment and can easily be administered with minimal training. To assess firefighters' physiological responses during the simulated functional tasks, we used the Zephyr BioHarness, a commercially available physiological monitoring device. The psychometric properties of the device have been established in the literature, and the device was deemed as reliable and valid. We also have very low precision regarding the effects of sex on our multiple regression analyses due to our male to female ratio of 15 : 1.

## 5. Conclusions

Hose drag and stair climb with a high-rise pack tasks were identified as physiological demanding tasks. Higher levels of cardiorespiratory fitness were associated with higher task performance, while higher body strength levels were only associated with higher hose drag task performance. Furthermore, age, sex, resting heart rate, and upper body/lower body strength levels had similar and moderate predictive values on hose drag and stair climb with a high-rise pack task completion times. Our findings imply that incorporating aerobic and strength exercises in firefighting training programs may potentially improve firefighting task performance.

## Figures and Tables

**Table 1:** Firefighters' demographic characteristics.

Demographics	Males	Females	All
Sample size	46.00	3.00	49.00
Age (years)	33.48 ± 9.42	36.00 ± 5.00	33.66 ± 9.19
Height (m)	1.82 ± 0.072	1.69 ± 0.05	1.81 ± 0.08
Weight (kg)	91.61 ± 12.60	71.00 ± 5.20	90.35 ± 13.22
Body mass index (kg/m^2^)	27.71 ± 3.54	24.86 ± 3.11	27.53 ± 3.56
Resting heart rate (bpm)	73.76 ± 10.78	76.67 ± 10.07	73.94 ± 10.66
85% heart rate-max (bpm)	158.52 ± 8.04	156.33 ± 4.50	158.40 ± 7.85
VO_2max_ (ml/kg/min)	40.54 ± 6.38	36.70 ± 1.17	40.30 ± 6.25
NIOSH lower limb strength (kg)	140.48 ± 26.70	107.00 ± 26.51	138.43 ± 27.63
Grip strength, right (kg)	60.49 ± 9.41	42.40 ± 9.07	59.38 ± 10.28
Grip strength, left (kg)	57.65 ± 9.17	38.43 ± 8.22	56.47 ± 10.17
Combined grip strength (kg)	118.14 ± 17.60	80.833 ± 16.07	115.85 ± 19.56

**Table 2:** Firefighters' physiologic responses and task completion times.

Variable	*n*	Mean	SD	Max	Min
Heart rate at hose drag (bpm)	49	163.00	16.00	195.00	125.00
Respiratory rate at hose drag (brpm)	49	27.00	4.00	40.00	23.00
HR-max% at hose drag (HR-max%)	49	88.00	8.00	106.00	64.00
Rating of perceived exertion hose drag (0–10)	49	1.78	1.10	5.00	1.00
Time elapsed to complete hose drag (seconds)	49	59.00	15.00	100.00	33.00
Heart rate at stair climb (bpm)	49	166.00	13.00	197.00	137.00
Respiratory rate at stair climb (brpm)	49	31.00	4.00	41.00	25.00
HR-max% at stair climb (%)	49	89.00	7.00	102.00	69.00
Rating of perceived exertion stair climb (0–10)	49	2.70	1.40	6.00	1.00
Time elapsed to complete stair climb (seconds)	49	59.00	14.50	93.00	30.00

**Table 3:** Intercorrelations among firefighters' fitness parameters and individual task completion times.

Variable	Hose drag (*r*)	Stair climb (*r*)
VO_2max_ (ml/kg/min)	−0.30^*∗∗*^	−0.31^*∗∗*^
NIOSH lower limb strength (kg)	−0.20^*∗∗*^	0.20
Combined grip strength (kg)	−0.20^*∗∗*^	0.10
Left grip strength (kg)	−0.10^*∗∗*^	0.10
Right grip strength (kg)	−0.25^*∗∗*^	0.10

^*∗∗*^ *p* < 0.05.

**Table 4:** Regression model for factors predicting hose drag completion times.

Label	Coefficient	SE	*p*	Part-squared	Model *r*^2^	Model SE
Model 1						
Intercept	26.51	22.70	-	-	0.24	13.55
Age	0.48	0.23	0.03	0.081		
Right grip strength	−0.77	0.35	0.03	0.086		
Left grip strength	0.54	0.36	0.13	0.042		
Sex	5.23	9.10	0.57	0.005		
Resting HR	0.36	0.19	0.065	0.064		

SE: standard error.

**Table 5:** Regression model for factors predicting stair climb with high-rise pack completion times.

Label	Coefficient	SE	*p*	Part-squared	Model *r*^2^	Model SE
Model 1						
Intercept	0.13	21.00	-	-	0.25	13.10
NIOSH	0.21	0.07	0.005	0.147		
Age	0.46	0.22	0.030	0.077		
Sex	−10.80	8.22	0.190	0.029		
Resting HR	0.33	0.18	0.070	0.055		

SE: standard error.

## Data Availability

The data used to support the findings of this study are available from the corresponding author upon request.

## References

[1] Braedley S. (2015). Pulling men into the care economy: The case of Canadian firefighters. *Competition and Change*.

[2] Selkirk G. A., McLellan T. M. (2004). Physical work limits for Toronto firefighters in warm environments. *Journal of Occupational and Environmental Hygiene*.

[3] Ensari I., Motl R. W., Klaren R. E., Fernhall B., Smith D. L., Horn G. P. (2017). Firefighter exercise protocols conducted in an environmental chamber: developing a laboratory-based simulated firefighting protocol. *Ergonomics*.

[4] Fahy R. F., LeBlanc P. R., Molis J. L. (2016). Firefighter Fatalities in the United States-2015. *National Fire Protection Association*.

[5] Poplin G. S., Roe D. J., Peate W., Harris R. B., Burgess J. L. (2014). The association of aerobic fitness with injuries in the fire service. *American Journal of Epidemiology*.

[6] Perroni F., Guidetti L., Cignitti L., Baldari C. (2014). Psychophysiological responses of firefighters to emergencies: A review. *The Open Sports Sciences Journal*.

[7] Michaelides M. A., Parpa K. M., Henry L. J., Thompson G. B., Brown B. S. (2011). Assessment of physical fitness aspects and their relationship to firefighters' job abilities. *The Journal of Strength and Conditioning Research*.

[8] Perroni F., Cortis C., Minganti C., Cignitti L., Capranica L. (2013). Maximal oxygen uptake of Italian firefighters: Laboratory vs. field evaluations. *Sport Sciences for Health*.

[9] Kales S. N., Soteriades E. S., Christophi C. A., Christiani D. C. (2007). Emergency duties and deaths from heart disease among firefighters in the United States. *The New England Journal of Medicine*.

[10] Canadian Society for Exercise Physiology (1998). *The Canadian Physical Activity, Fitness and Lifestyle Appraisal*.

[11] Waters T. R., Putz-Anderson V., Garg A., Fine L. J. (1993). Revised NIOSH equation for the design and evaluation of manual lifting tasks. *Ergonomics*.

[12] Weller I. M. R., Corey P. N. (1998). A study of the reliability of the Canada fitness survey questionnaire. *Medicine & Science in Sports & Exercise*.

[13] Macdermid J. C., Alyafi T., Richards R. (2001). Test-retest reliability of static and endurance grip strength tests performed on the Jamar and NK. *Physiotherapy Canada*.

[14] Zephyr Technology BioHarness 3.0 User ManualBioHarness 3.0 User Manual. http://zephyranywhere.com/products/bioharness-3/2012.

[15] Jeong T.-S., Reilly T., Morton J., Bae S.-W., Drust B. (2011). Quantification of the physiological loading of one week of “pre-season” and one week of “in-season” training in professional soccer players. *Journal of Sports Sciences*.

[16] Williams-Bell F. M., Villar R., Sharratt M. T., Hughson R. L. (2009). Physiological demands of the firefighter candidate physical ability test. *Medicine & Science in Sports & Exercise*.

[17] Sheaff A. K., Bennett A., Hanson E. D. (2010). Physiological determinants of the Candidate Physical Ability Test in firefighters. *The Journal of Strength and Conditioning Research*.

[18] Evans J. D. (1996). *Straightforward Statistics for the Behavioral Sciences*.

[19] Perroni F., Tessitore A., Lupo C., Cortis C., Cignitti L., Capranica L. (2008). Do Italian fire fighting recruits have an adequate physical fitness profile for fire fighting?. *Sport Sciences for Health*.

[20] Myhre L. G., Tucker D. M., Bauer D. H. Relationship between selected measures of physical fitness and performance of a simulated fire fighting emergency task. http://www.dtic.mil/docs/citations/ADA319915.

[21] Rhea M. R., Alvar B. A., Gray R. (2004). Physical fitness and job performance of firefighters. *The Journal of Strength and Conditioning Research*.

[22] Markovic G. (2007). Poor relationship between strength and power qualities and agility performance. *The Journal of Sports Medicine and Physical Fitness *.

[23] Young W. B., James R., Montgomery I. (2002). Is muscle power related to running speed with changes of direction?. *The Journal of Sports Medicine and Physical Fitness *.

[24] Badley E. M., Crotty M. (1995). An international comparison of the estimated effect of the aging of the population on the major cause of disablement, musculoskeletal disorders. *The Journal of Rheumatology*.

[25] Sluiter J. K., Frings-Dresen M. H. W. (2007). What do we know about ageing at work? Evidence-based fitness for duty and health in fire fighters. *Ergonomics*.

[26] Sinden K., MacDermid J., Buckman S., Davis B., Matthews T., Viola C. (2013). A qualitative study on the experiences of female firefighters. *Work*.

